# Safely prolonging single breath-holds to >5 min in patients with cancer; feasibility and applications for radiotherapy

**DOI:** 10.1259/bjr.20160194

**Published:** 2016-07

**Authors:** Michael J Parkes, Stuart Green, Andrea M Stevens, Sophia Parveen, Rebecca Stephens, Thomas H Clutton-Brock

**Affiliations:** ^1^ School of Sport, Exercise and Rehabilitation Sciences, University of Birmingham, Birmingham, UK; ^2^ National Institute for Health Research (NIHR)/Wellcome Trust Birmingham Clinical Research Facility, Queen Elizabeth Hospital, Birmingham, UK; ^3^ Hall Edwards Radiotherapy Group, University Hospitals Birmingham NHS Foundation Trust, Queen Elizabeth Hospital, Birmingham, UK; ^4^ Department Anaesthesia and Intensive Care Medicine, University of Birmingham and University Hospitals Birmingham NHS Foundation Trust, Queen Elizabeth Hospital, Birmingham, UK

## Abstract

**Objective:**

Multiple, short and deep inspiratory breath-holds with air of approximately 20 s are now used in radiotherapy to reduce the influence of ventilatory motion and damage to healthy tissue. There may be further clinical advantages in delivering each treatment session in only one single, prolonged breath-hold. We have previously developed techniques enabling healthy subjects to breath-hold for 7 min. Here, we demonstrate their successful application in patients with cancer.

**Methods:**

15 patients aged 37–74 years undergoing radiotherapy for breast cancer were trained to breath-hold safely with pre-oxygenation and mechanically induced hypocapnia under simulated radiotherapy treatment conditions.

**Results:**

The mean breath-hold duration was 5.3 ± 0.2 min. At breakpoint, all patients were normocapnic and normoxic [mean end-tidal partial pressure of carbon dioxide was 36 ± 1 standard error millimetre of mercury, (mmHg) and mean oxygen saturation was 100 ± 0 standard error %]. None were distressed, nor had gasping, dizziness or disturbed breathing in the post-breath-hold period. Mean blood pressure had risen significantly from 125 ± 3 to 166 ± 4 mmHg at breakpoint (without heart rate falling), but normalized within approximately 20 s of the breakpoint. During breath-holding, the mean linear anteroposterior displacement slope of the *L* breast marker was <2 mm min^−1^.

**Conclusion:**

Patients with cancer can be trained to breath-hold safely and under simulated radiotherapy treatment conditions for longer than the typical beam-on time of a single fraction. We discuss the important applications of this technique for radiotherapy.

**Advances in knowledge:**

We demonstrate for the first time a technique enabling patients with cancer to deliver safely a single prolonged breath-hold of >5 min (10 times longer than currently used in radiotherapy practice), under simulated radiotherapy treatment conditions.

## INTRODUCTION

Respiratory-related motion of 2–3 cm^1^ remains a major problem in radiotherapy planning and delivery for most thoracic and abdominal cancers. Such motion requires substantial enlargement (by 1 cm or more) in the planned treatment field and encroachment on vital structures. This requires management that will be especially important with the imminent introduction of MR-guided radiotherapy.^2^ Koybasi et al^3^ report that for irregular breathing patterns, four-dimensional CT may inaccurately characterize tumour motion and location, with negative consequences particularly for treatment delivered with scanned proton beams. Furthermore, the correspondence between breathing motion assessed by four-dimensional CT at the planning stage and the motion during each daily treatment is rarely measured in routine clinical practice.

Gating methodologies^4^ appear to be accurate, but increase the duration of treatment slots. Tracking is technically challenging, as the latencies of critical systems such as the multileaf collimator and associated control systems are such that a predictive model of linear motion is required for accurate tracking.^5^ Specialized techniques such as CyberKnife^®^ (Accuray Inc., Sunnyvale, CA) measure this correspondence well, allowing tracking during treatment by a combination of dynamic monitoring of the chest surface contours with regular planar X-ray imaging of internal anatomy,^6^ and other approaches to tracking are developing. Particle therapy introduces the additional challenge of range changes, for instance should a tracked lung lesion move in and out of the shadow of a rib.^2^

Currently, such motion can be addressed to a limited extent by asking patients to breathe to a metronome or to achieve inflation volumes within visible guidelines.^1,7,8^ But, all such techniques depend on how well patients respond to feedback and continue to comply with instructions. Newly emerging techniques involve using multiple, short and deep inspiratory breath-holds with air of approximately 20 s.^1,9–13^ There is, however, a simple and practical alternative: to deliver each treatment in a single prolonged breath-hold.

The basic physiology of breath-holding is well understood.^14,15^ But, until recently, this knowledge had no clinical application and is barely taught in medicine. For the last 5 years, >3 articles per week in radiotherapy journals describe the application of multiple short breath-holds without appreciating the advantages this physiological knowledge can already offer. First, the combination of training, practice, pre-oxygenation and mechanically induced hypocapnia^16^ is already known to achieve mean single breath-hold durations of 7 min in healthy and experienced volunteers^17^ and of >5 min in inexperienced volunteers.^18^ Secondly, it is straightforward to incorporate limits with readily available and non-invasive monitoring equipment^18^ to ensure patient safety against the two principal risks of prolonged breath-holds, asphyxia and raised blood pressure. Thirdly, the cause of the slight chest deflation during breath-holding is already understood^14^ and should have a small, linear and predictable effect on chest motion.

We have used this knowledge to establish for the first time whether patients with cancer undergoing radiotherapy can be trained to sustain a single prolonged breath-hold that is long enough to deliver a single and typical radiotherapy beam-on time (approximately 2 min). We tested whether they could deliver it under simulated radiotherapy treatment conditions. We also measured the three-dimensional movement of the chest surface during the single prolonged breath-hold.

## METHODS AND MATERIALS

### Patient recruitment

Conduction of experiments followed the Declaration of Helsinki and approval of the local research ethics committee. In we trained 15 female patients with breast cancer aged 37–74 years (mean age 54 years, with 4 patients aged 60 years or above) all undergoing radiotherapy (Table 1), with 12 patients having received chemotherapy (11 patients of whom had anthracycline-based chemotherapy and 2 patients received trastuzumab). None had respiratory, cardiovascular or neurological disease, diabetes or obesity. All were non-smokers with no previous experience of breath-holding.

**Table 1.** Treatment with chemotherapy and trastuzumab during the breath-holding study

Subjects	Chemotherapy	Days before breath-hold when last chemotherapy given	Trastuzumab treatment during breath-hold
1	FEC-T	124	Trastuzumab
2	FEC-T	134	None
3	ECMF	26	None
4	ECMF	136	None
5	FEC-T	67	None
6	FEC-T	41	None
7	FEC-T	96	None
8	FEC-T	107	None
9	FEC-T	180	None
10	None	*	None
11	FEC-T	159	None
12	None	*	None
13	None	*	None
14	Taxotere/cyclophosphamide	50	None
15	FEC-T	138	Trastuzumab

C, cyclophosphamide; E, epirubicin; F, 5 flurouracil; M, methotrexate; T, docetaxel.

*indicates not applicable.

### Initial instruction and training

**Figure 1.**
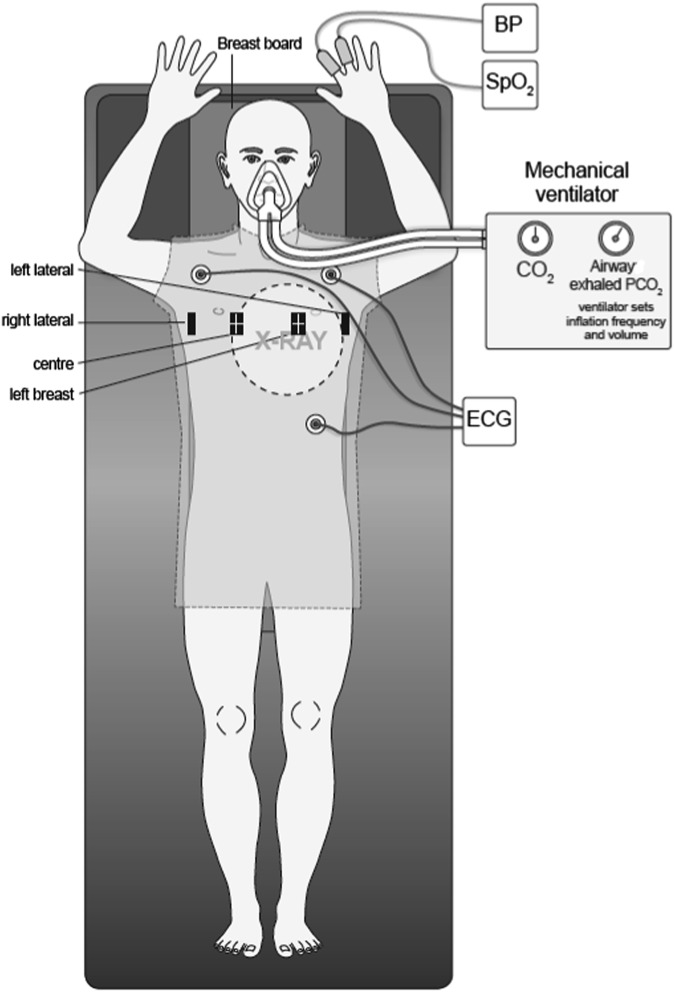
Equipment on the patient during mechanical ventilation in the simulator room. BP, blood pressure; ECG, electrocardiography; PCO_2_, partial pressure of carbon dioxide; SpO_2_, oxygen saturation. Reproduced from Parkes et al^19^ with permission from The British Institute of Radiology.

**Figure 2.**
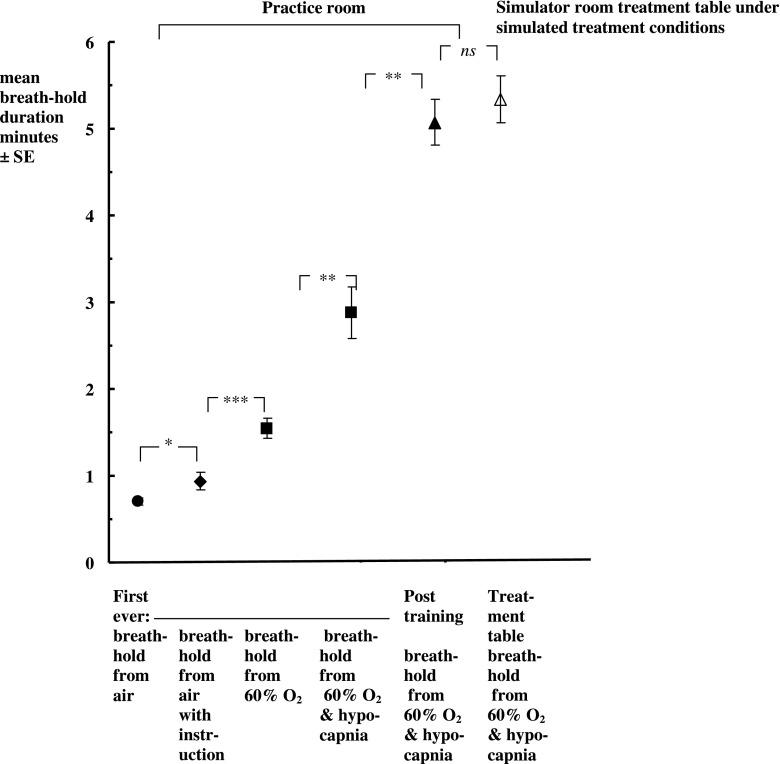
Mean breath-hold durations of >5 min in 15 patients with breast cancer. ns *p*-value > 0.05, *p-value < 0.05, **p-value < 0.01, ***p-value < 0.001 by paired *t* test.

On the first session (Day 1), they lay at rest on a bed and were instrumented for safety^17−19^ to measure the blood pressure (Finapres 2300 Ohmeda, Englewood, CA), chest electrocardiography (Lead I) and oxygen saturation (SpO_2_; finger pulse oximeter). Figure 1 summarizes the instrumentation after the patients had been connected to the mechanical ventilator (as described below). Patients were deliberately not told how long they might be expected to breath-hold, nor how long others were holding. Patients did not know when the breath-hold would start, were not allowed to see a clock during breath-holding and listened to music throughout. We followed the safety limits we described previously,^18^ with patients being instructed to break their breath-hold if their systolic blood pressure (sBP) consistently exceeded 180 mmHg and/or SpO_2_ fell below 94%. Table 2 shows the order of the experimental conditions (and their resulting breath-holds are given in Figure 2). For their first ever breath-hold with air (Table 2, order 1), patients breathed freely and were merely asked to breath-hold as long as possible. This was measured with a stopwatch.

**Table 2.** Order of experimental conditions

Order	Condition	Session
1	First ever breath-hold, with a maximum inflation with air	1
2	Instruction and training in breath-hold technique; patient connected to mechanical ventilator but only on the spontaneous setting, inhale, exhale, inhale maximally and then breath-hold from air after instruction	1
3	As (2) above, but after breathing 60% oxygen for 4 min, then breath-hold from 60% oxygen	1
4	As (3) above, but after 15 min of mechanical ventilation-induced hypocapnia with 60% oxygen, then breath-hold from 60% oxygen and hypocapnia	1
5	As (4) above, repeated as necessary for post-training breath-hold from 60% oxygen and hypocapnia	2–4
6	As (5) above, repeated in simulator room under simulated treatment conditions, then treatment table breath-hold from 60% oxygen and hypocapnia	5

All were connected *via* a facemask to a Draeger Evita 2 ventilator (Drager, Lubeck, Germany) and familiarized to spontaneous breathing on the ventilator (on its assisted spontaneous breathing setting) and to being mechanically hyperventilated by the ventilator (on its intermittent positive-pressure ventilation setting).^16−20^

They were then instructed on how best to maintain a relaxed posture, to inhale, exhale and inhale air maximally and to breath-hold by closing their mouth and pharyngeal valve. Nose clips were not used. They were taught not to attempt pushing or inhaling against a closed glottis and this was easily confirmed^17,21^ by observation of their chest and neck and of their blood pressure record during experiments. They were told that they were safe to try breath-holding longer, as their safety was continuously monitored and they would be instructed to stop if they reached our safety limits. To measure the breath-hold duration objectively from airway pressure and the partial pressure of carbon dioxide in the expired gas, we used the disposable facemask with a pressure transducer (Digitimer Ltd, Hertfordshire, UK) and an in-line capnograph (Hewlett–Packard 78536A capnograph; Hewlett Packard^®^, Palo Alto, CA). End-tidal partial pressure of carbon dioxide (PetCO_2_) measurements confirmed that they did not surreptitiously hyperventilate before breath-holding. PetCO_2_ and mask pressure measurements also ensured that they did not take surreptitious breaths during breath-holding.

### Mechanical ventilation on spontaneous and positive-pressure ventilation settings

After initial training, patients breathed air spontaneously through the facemask connected to the ventilator and this was recorded for a 4-min resting period. They then inhaled air maximally, exhaled maximally and then inhaled maximally to breath-hold as long as possible (Table 2, order 2). Following recovery (about 2 min), they breathed 60% oxygen spontaneously through the facemask connected to the ventilator. This is the maximum percentage for pre-oxygenation that may safely be given for breath-holding without increasing the risk of atelectasis.^18^ After 4 min of this, they breath-held again as long as possible (Table 2, order 3). After recovery, they were then mechanically hyperventilated through the facemask with positive pressure in 60% oxygen at a 16–17 breaths per minute rhythm and at a tidal volume (approximately 1.2 l depending on their size) to induce hypocapnia. We maintained hypocapnia at the mean PetCO_2_ level of 20 ± 0 mmHg (the lowest level of hypocapnia that can be safely induced that causes paraesthesiae without causing hypocapnic tetany).^16,20,22,23^ After 15 min of hypocapnia, mechanical hyperventilation was switched off and patients were instructed to inhale 60% oxygen maximally, exhale maximally, inhale maximally and then breath-hold (Table 2, order 4). Only at the end of the day were patients told how long they had breath-held in each session. Patients were thoroughly debriefed at the end of each day to discuss what they felt and to reach agreement on what could be performed the next day to improve their performance.

On subsequent sessions (approximately Days 2–4), we practised only the breath-hold with pre-oxygenation and hypocapnia until we and the patients felt it had stabilized at the maximum duration with which each was comfortable (Table 2, order 5).

In the final session (Day 5), patients went to a simulator room in the Department of Radiotherapy containing an Osiris surface image-mapping system (Qados Ltd, Berkshire, UK) and the same equipment.^19^

They listened to music, wore a gown and lay on a breast board as shown in Figure 1 to mimic every aspect of radiotherapy (except for turning on the beam). Osiris markers were fixed on the gown in the following positions: right lateral, chest centre, left lateral and left breast. We chose not to fix the markers directly on their skin to preserve patient modesty and to ensure that they remained as relaxed as possible. Thenceforth, the marker positions were recorded continuously to derive their *x*, *y* and *z* positions to the nearest 0.1 mm.^19^ We then repeated the breath-hold following pre-oxygenation and hypocapnia (Table 2, order 6).

All physiological data were sampled at 2 kHz and recorded, stored and analyzed using a CED1401 data acquisition system (Cambridge Electronics Design, Cambridge, UK). The timing of the Osiris data recordings was synchronized offline with all other physiological data recordings. We described previously^18^ how we derived instantaneous heart rate, systolic, diastolic, mean pressure and SpO_2_ and resampled data to align the data correctly in all patients at the same time relative to the start and end of breath-holding.

### Statistical analysis

Statistical analysis was performed with paired comparisons within patients and unpaired comparisons with the data from our healthy subjects^18^ using analysis of variance for repeated comparisons or Student's *t-*test for single comparisons. Significance was taken at *p*-value < 0.05 for two-tailed tests, and means are given with ±standard error of the mean. Detailed mathematical analysis of the blood pressure changes between 20 and 100% of breath-holds used least-squares linear regression analysis. We assessed the adequacy (correlation coefficient squared; r^2) of a straight line fit or whether fitting two lines was significantly better than fitting one line and have expressed slopes as millimetre of mercury per minute (at percentage times of the mean breath-hold duration).

## RESULTS

### Breath-hold durations

Figure 2 shows the mean breath-hold durations of all patients. On Day 1 (Table 2, row 1), all could hold initially for 42 ± 2 s (and none <29 s). Instruction (Table 2, row 2) significantly increased the mean breath-hold duration with air to 58 ± 6 s. Pre-oxygenation significantly increased the mean breath-hold duration to 1.6 ± 0.1 min and adding hypocapnia increased it to 2.9 ± 0.3 min.

Performance significantly and progressively improved with practice on subsequent days, with the longest breath-hold in the practice room category reaching a mean of 5.1 ± 0.2 min. Even the eldest patient (74 years) held for 5.3 min.

Such breath-holds were safe. During training, none consistently reached our safety limits (two patients reached the SpO_2_ limit each on only one occasion, one of whom reaching the sBP limit on a different occasion). At breakpoint, none were distressed, nor experienced dizziness or disturbed breathing. All were undergoing radiotherapy and there was no detectable effect on the breath-hold duration of previous chemotherapy or current trastuzumab treatment (Table 1).

**Figure 3.**
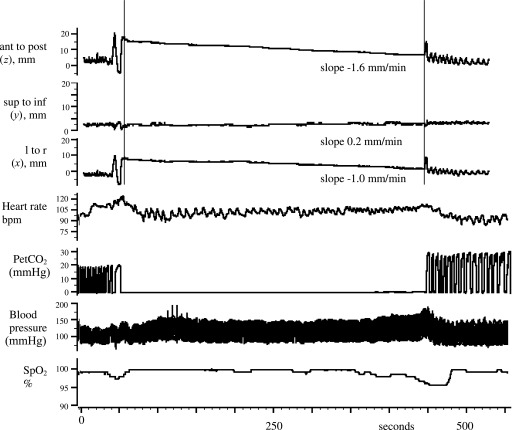
Polygraph of the longest breath-hold (6.6 min) under simulated treatment conditions. The left breast marker movement is indicated as left to right (l to r), superior to inferior (sup to inf) and anterior to posterior (ant to post). The slope is calculated as indicated between the vertical lines. bpm, beats per minute; mmHg, millimetre of mercury; PetCO_2_, end-tidal partial pressure of carbon dioxide; SpO_2_, oxygen saturation.

Figure 3 shows the complete polygraph record for the subject (aged 52 years) with the longest breath-hold (6.6 min) in the simulator room. This patient broke spontaneously just as she reached the systolic pressure safety limit, whereas SpO_2_ was still 97% (and PetCO_2_ was 30 mmHg). Figure 3 also shows that there is some settlement in the anteroposterior position of the left breast over the first approximately 15 s of the breath-hold, before the drift follows a linear trajectory.

Figure 2 shows that patients could reproduce their longest breath-holds while on the breast board in the simulator room (a mean of 5.3 ± 0.2 min). Under treatment conditions in the simulator room breath-holds, patients did not consistently reach the safety limits. In the simulator room, one patient reached the SpO_2_ limit and two patients reached the systolic pressure limit (none having previously reached these limits during training). During all breath-holds, the chest deflates slightly. Table 3 shows that during breath-holding, the mean movement of all markers in the anteroposterior (*z*) direction was <2.2 mm min^−1^.

**Table 3.** Slight chest deflation during breath-holding

Anteroposterior (*z*) movement of	Mean mm min^−1^	SE mm min^−1^	*n*
Left breast marker	−1.9	0.3	15
Chest marker	−2.1	0.3	11
Left lateral marker	−1.1	0.3	14
Right lateral marker	−1.1	0.2	14
Mean superior/inferior (*y*) movement of all markers	0.1	0.1	11
Mean left/right (*x*) movement of all markers	−0.6	0.2	11

SE, standard error.

### Patient physiological responses at rest and during breath-holding

During the 4-min rest period and while breathing air, the patient's mean sBP was 131 ± 5 mmHg, averaged mean pressure was 83 ± 2 mmHg, diastolic pressure was 59 ± 2 mmHg, mean heart rate was 75 ± 2 beats per minute (bpm), mean PetCO_2_ level was 32 ± 1 mmHg and mean SpO_2_ was 99 ± 0%. Pre-oxygenation lowered the resting heart rate by 2 bpm (*p*-value < 0.05) and this with hypocapnia raised the resting heart rate by 4 bpm (*p*-value < 0.05), but these had no significant effects on the resting blood pressure. We found similarly small or no such changes in the resting heart rate and blood pressure in previous studies with pre-oxygenation and hypocapnia.^16−18,20^

**Figure 4.**
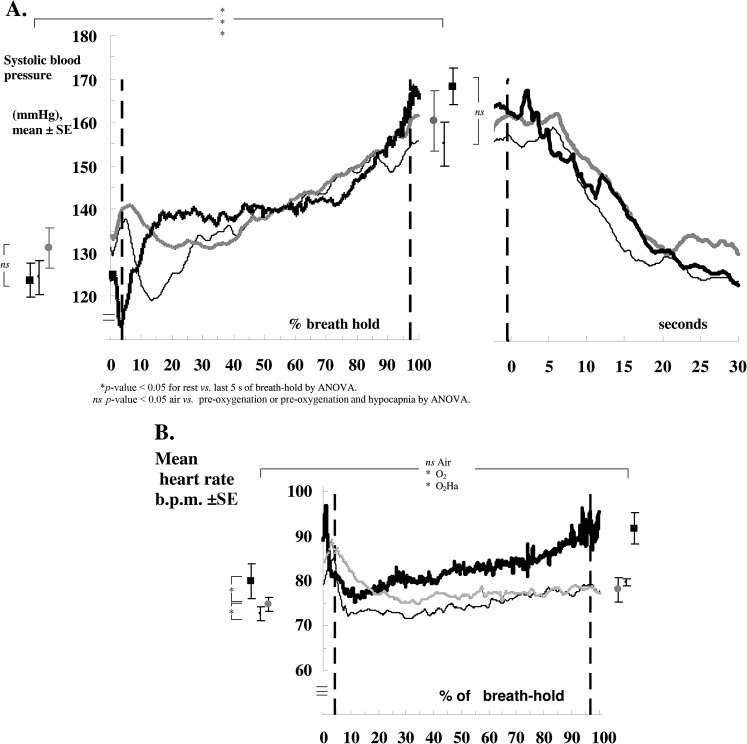
Blood pressure rises linearly (without heart rate falling) during breath-holding in 15 patients: (a) mean systolic blood pressure. (b) Mean heart rate lines refer to breath-holds with air (grey line), pre-oxygenation (thin black line) and pre-oxygenation with hypocapnia (thick black line). ns *p*-value > 0.05, *p-value < 0.05 by analysis of variance (ANOVA) and paired *t* test. mmHg, millimetre of mercury; SE, standard error.

Figure 3 shows the cardiovascular changes in one patient. Figure 4a shows that breath-holding caused significant increases in mean sBP (reaching for air 160 ± 7 mmHg, for pre-oxygenation 155 ± 5 and for pre-oxygenation with hypocapnia 168 ± 4 mmHg). The failure of the heart rate to fall (Figure 4b) confirms that the heart rate component of the baroreflex is attenuated during breath-holding.^18^ Indeed at breakpoint, the mean heart rate actually rose slightly from resting levels, by 6 bpm (*p*-value < 0.05) for breath-holds with pre-oxygenation and by 12 bpm (*p*-value < 0.05) for breath-holds with pre-oxygenation and hypocapnia (Figure 4b).

Furthermore, Figure 4 shows that the mean pressure rise was the same (*i.e.* it was not abolished) with pre-oxygenation and hypocapnia, confirming that the pressure rise is not caused by a systemic chemoreflex.^18^ Figure 4 shows that the mean systolic pressure always returned to resting levels within 20 s of the breakpoint.

### Similarity of patients' physiological responses to those of healthy untrained subjects

The mean resting heart rate of patients was not significantly different from that of our healthy untrained subjects,^18^ although the mean sBP of the patients was lower (by 3–12 mmHg, *p*-value < 0.05) and the mean resting PetCO_2_ level was lower (by 6 mmHg, *p*-value < 0.001).

After sufficient practice with pre-oxygenation and hypocapnia, patients' mean breath-hold duration was not significantly shorter than that in our untrained normal subjects—5.5 ± 0.5 min.^18^

At the breakpoint for breath-holds with air, patients had similar systolic pressure levels, sPO_2_ levels (reaching a nadir of 98 ± 1% at 15 s post breakpoint) and PetCO_2_ levels (to 44 ± 1 mmHg). Patients did, however, have a slightly higher mean heart rate at breakpoint (by 7 bpm, *p*-value < 0.05).

At breakpoint from pre-oxygenation and hypocapnia, the mean blood pressure and heart rate were higher in patients (by 15 mmHg, *p*-value < 0.05, and by 12 bpm, *p*-value < 0.05) than in healthy subjects.

Detailed mathematical analysis of the course of the blood pressure rises during breath-holding in both patients (Figure 4a) and healthy volunteers in our previous study^18^ showed that throughout breath-holds with air and with oxygen, the better fit for both was a small but linear rise in pressure (for patients, the air slope was 7 mmHg min^−1^ and the oxygen slope was 6 mmHg min^−1^ and for normal subjects, the air and oxygen slopes were both 6 mmHg min^−1^). Whereas, for breath-holds with pre-oxygenation and hypocapnia, the better fit for both was a small linear rise for the first 75% of the breath-hold (patient slope of 1 mmHg min^−1^ and normal subject slope of 8 mmHg min^−1^), followed by a steeper rise over the last 25% of the breath-hold (patient slope of 20 mmHg min^−1^ and normal subject slope of 18 mmHg min^−1^).

## DISCUSSION

We show for the first time that patients with cancer undergoing radiotherapy can be trained to deliver safely and under treatment conditions a single breath-hold of >5 min. This is >10 times longer than currently used in radiotherapy practice. Our technique may offer opportunities to improve imaging and radiotherapy delivery for a wide range of thoracic and abdominal tumours.

### Breath-hold durations

The >5-min breath-hold durations we achieved in patients with breast cancer are not significantly different from those we achieved in similarly inexperienced and much younger healthy volunteers.^18^ Much longer durations have been achieved by us^17^ and others,^23^ before safety was such an overt consideration. Indeed we have achieved breath-hold durations of up to 12 min in some healthy volunteers that are still within our safety limits [MJ Parkes et al, personal communication]. Further research is still required to understand how some but not all achieve this, and such research might benefit patients who are more anxious or have compromised lung function.

Success appears to be due to the combination of training, practice, pre-oxygenation and mechanically induced hypocapnia, rather than any one particular element. Others^24^ used our basic methodology but with voluntary hyperventilation and achieved mean durations in patients with breast cancer of 2.8 min. The advantage of patients voluntarily hyperventilating is that it requires less equipment; but, the disadvantage is that the level of hypocapnia is much harder to control. Furthermore, voluntary hyperventilation requires much concentration and physical effort from patients. Voluntary hyperventilation in combination with the above elements may well have clinical application, if routine single and prolonged breath-holds of under approximately 3-min duration are required. But, we believe that most patients will find voluntary hyperventilation too difficult, whereas we know they find mechanical hyperventilation straightforward.^19^ This fact, in combination with the time required for final positioning, staff leaving the room and beam delivery checks for setup, indicates to us that if single and prolonged breath-holds have widespread application in radiotherapy, resources would be best applied to develop mechanical rather than voluntary hyperventilation. Furthermore, single prolonged breath-holds would have no detrimental effects on clinical efficiency, since all training could be achieved by non-radiotherapy staff at a separate location for as long as necessary for each patient. This is of particular importance in very high-cost applications such as proton and particle beam radiotherapy, where time spent in the treatment room is at a premium. With our technique, only when fully trained would patients attend the radiotherapy department.

For routine clinical delivery of single prolonged breath-holds, we therefore recommend developing a simplified ventilator for radiotherapy use together with non-invasive measurement of PetCO_2_ to control the depth of hypocapnia achieved. Our recommendations^18^ for patient safety during breath-holding should be followed, requiring non-invasive equipment to measure SpO_2_ and sBP.

### Linear chest movement during single and prolonged breath-holds

The chest normally deflates slightly (by approximately 300 ml min^−1^)^25^ during all breath-holds because metabolism results in gaseous oxygen continuing to be extracted from alveolar air, whereas an equal volume of gaseous carbon dioxide cannot escape from blood into alveolar air to replace it.^14^ Since there is no reason for the metabolic rate to fluctuate during breath-holding, the rate of this deflation should be linear. Our direct measurements establish that it is. The largest deflation movement (<2.2 mm min^−1^) is in the anteroposterior direction, but we may have overestimated all such movements, first because our markers were placed on the gown rather than on the skin and secondly because our drift measurement includes the initial settlement in position over the first approximately 15 s of the breath-hold. The size of this linear deflation movement during single prolonged breath-holds is far smaller than the rhythmic 2–3 cm of movement measured during breathing in radiotherapy studies.^1^ It should be straightforward to predict the trajectory of this linear movement during treatment planning by measuring it when patients are asked to mimic it by slowly exhaling the same volume. Another possibility is to abolish this deflation movement altogether. This could be achieved by using the mechanical ventilator to reinflate the chest at a rate equivalent to the metabolic rate (rate of oxygen consumption) during the breath-hold.

### Patient recruitment and experience

With no previous experience of training any patients with cancer for single prolonged breath-holds, we initially believed that single and prolonged breath-holds would be achieved only in patients who are relatively young, fit and exercise conscious.

We were not able to recruit such patients. Instead, we were only able to recruit 15 older patients (a mean age of 54 years). But, we found that breath-hold duration was not obviously related to age. We were cautious with our first few patients and were satisfied with 3–4-min breath-holds. With the experience gained with later patients, however, we now believe we could have trained the first patients to hold even longer.

Similarly, with no previous experience, we devised rigorous exclusion criteria, had trained resuscitation staff available and set cautious safety limits for breath-hold termination. We could now relax some of these exclusion criteria to recruit a more representative range of the patient population. But, we would still retain the initial health screening and we would not withdraw our safety limits.

All patients were volunteers, motivated to help other patients, but the experience gained would now enable us to involve more reserved patients. All continued with radiotherapy during our training and while 12 patients also had had chemotherapy and 2 patients were receiving trastuzumab, these had no effect on breath-hold duration. We saw no evidence that patient performance decreased over time, while radiotherapy treatment continued. Indeed, we feel we gave our patients relatively little training. We would not be surprised if breath-hold durations continued to improve as they become part of multiple treatment sessions.

### Wider applications for radiotherapy practice

While our pilot study focused on patients with breast cancer, the results will be representative of all patients with normal lung function. Our approach could therefore be of benefit to patients with tumours in a wide range of thoracic and abdominal locations including the lung, oesophagus, liver, pancreas, stomach and kidney. Management of respiratory-related motion at all these sites is the subject of urgent ongoing research and technical modifications. Our pilot data indicate that our novel approach should be brought into this debate.

For patients with varying degrees of compromised lung function, there is a particular need for investigating their tolerance of our technique. But, patients with all levels of compromised lung function are routinely ventilated while under general anaesthesia, as dictated by clinical needs. Moreover, when conscious, these patients should find mechanical ventilation more comfortable than spontaneous breathing, because mechanical ventilation abolishes the effort of breathing. Our next focus therefore is on the feasibility of our technique in patients with compromised lung function.
